# American Journal of Opthalmology

**Published:** 1862-07

**Authors:** 


AMERICAN JOURNAL OF OPTHALMOLOGY. Vol. 1., No. 1. By JULIUS HOMBERGER, M.D., Editor and Proprietor. New York.

We have received the first number of this new Journal, devoted wholly to the consideration of diseases of the eye. It is a Monthly Journal of 48 pages, printed on good paper, and in good style, by Bailliere Brothers, 440 Broadway, New York. We cheerfully welcome it to our exchange list, and commend it to the patronage of the profession.

